# Searching for Real-World Effectiveness of Health Care Innovations: Scoping Study of Social Prescribing for Diabetes

**DOI:** 10.2196/jmir.6431

**Published:** 2017-02-02

**Authors:** Karen Pilkington, Martin Loef, Marie Polley

**Affiliations:** ^1^ School of Life Sciences Faculty of Science and Technology University of Westminster London United Kingdom; ^2^ School of Health Sciences and Social Work Faculty of Science University of Portsmouth Portsmouth United Kingdom; ^3^ Institute of Transcultural Health Studies European University Viadrina Frankfurt Germany

**Keywords:** diabetes mellitus, type 2, evaluation studies, primary health care, program evaluation

## Abstract

**Background:**

Social prescribing is a process whereby primary care patients are linked or referred to nonmedical sources of support in the community and voluntary sector. It is a concept that has arisen in practice and implemented widely in the United Kingdom and has been evaluated by various organizations.

**Objective:**

The aim of our study was to characterize, collate, and analyze the evidence from evaluation of social prescribing for type 2 diabetes in the United Kingdom and Ireland, comparing information available on publicly available websites with the published literature.

**Methods:**

We used a broad, pragmatic definition of social prescribing and conducted Web-based searches for websites of organizations providing potentially relevant services. We also explored linked information. In parallel, we searched Medline, PubMed, Cochrane Library, Google Scholar, and reference lists for relevant studies published in peer-reviewed journals. We extracted the data systematically on the characteristics, any reported evaluation, outcomes measured and results, and terminology used to describe each service.

**Results:**

We identified 40 UK- or Ireland-based projects that referred people with type 2 diabetes and prediabetes to nonmedical interventions or services provided in the community. We located evaluations of 24 projects; 11 as published papers, 12 as Web-based reports, and 1 as both a paper and a Web-based report. The interventions and services identified included structured group educational programs, exercise referral schemes, and individualized advice and support with signposting of health-related activities in the community. Although specific interventions such as community-based group educational programs and exercise referral have been evaluated in randomized controlled trials, evaluation of individualized social prescribing services involving people with type 2 diabetes has, in most cases, used pre-post and mixed methods approaches. These evaluations report generic improvement in a broad range of outcomes and provide an insight into the criteria for the success of social prescribing services.

**Conclusions:**

Our study revealed the varied models of social prescribing and nonmedical, community-based services available to people with type 2 diabetes and the extent of evaluation of these, which would not have been achieved by searching databases alone. The findings of this scoping study do not prove that social prescribing is an effective measure for people with type 2 diabetes in the United Kingdom, but can be used to inform future evaluation and contribute to the development of the evidence base for social prescribing. Accessing Web-based information provides a potential method for investigating how specific innovative health concepts, such as social prescribing, have been translated, implemented, and evaluated in practice. Several challenges were encountered including defining the concept, focusing on process plus intervention, and searching diverse, evolving Web-based sources. Further exploration of this approach will inform future research on the application of innovative health care concepts into practice.

## Introduction

The World Health Organization global report on diabetes revealed that the number of people with diabetes had risen from 108 million in 1980 to 422 million in 2014 [1]. A similarly dramatic increase is anticipated in the future in the United Kingdom, as the number of people with type 2 diabetes is projected to increase by 50% between 2010 and 2030 [2]. Major advances have occurred in diabetes research, showing that lifestyle interventions can delay or prevent the onset of diabetes [3,4], but prevention and good control of diabetes remain elusive for most of the population [5]. The primary care sector represents a potentially important setting in this context, as more than 85% of the population in the United Kingdom visits a general practitioner (GP) at least once a year [6]. Furthermore, to increase the sustainability of general practice, other options for the support and care of patients, particularly those with chronic conditions, are being sought by organizations such as National Health Service (NHS) England [7].

Social prescribing (or “community referral”) is a relatively new approach in health care, aiming to create referral pathways that enable the GP or a health care practitioner to refer patients with social or practical needs to a local provider of nonclinical services [8,9]. These are often offered by volunteers or the community sector and cover a wide range of interventions including educational sessions, exercise training, dietary advice, creative activities, self-help groups, emotional or social support, and stress management. A current challenge is to systematically collate and evaluate the evidence of the impact of social prescribing on people’s lives, its conceptualization, dissemination, and the way it is operationalized in practice.

In general, the translation of research into practice encompasses multiple dimensions, with different levels of scientific involvement and routes of publication. It covers projects that analyze selected aspects of the implementation of new treatments or research knowledge into a practical setting to ensure they not only reach the patients for whom they are intended but are also implemented in the most effective manner [10]. The focus in research is frequently on the efficacy and generalizability of public health interventions, whereas there may be equal levels of interest in the local implementation of interventions in a real-world setting, with “scientific” outcomes being of minor interest, resulting in a low priority for publication. As a result, using the standards of and processes for conducting systematic reviews on health care interventions [11,12] may not represent an adequate approach for gathering evidence of translational research including real-world projects. Limited numbers of studies are available in peer-reviewed articles and, therefore, in the major databases for synthesizing data and drawing conclusions. However, although other sources such as evaluations in practice may be available, these may be limited in methodological and reporting quality and difficult to locate.

Previous reviews of the evidence on social prescribing have been reported [13,14] as have evaluations of schemes such as exercise on referral [15-17], which fit the concept of social prescribing. Innovative care programs have been evaluated, which include elements of social prescribing and encourage commissioning of nontraditional providers to support people with long-term conditions including diabetes [18], as have fitness programs involving various initiatives across the United Kingdom [19]. There has not, however, been an in-depth review focusing on social prescribing for a specific health area such as diabetes in a specific context (the United Kingdom) with a comparison of the data available from formal (published) and informal Web-based (gray literature) sources.

The aim of this study was to characterize social prescribing, and to then collate and analyze evaluations of social prescribing, by focusing on services or projects for people with type 2 diabetes in the United Kingdom and Ireland. This allowed an examination of how an innovative health care concept has been translated and applied in practice and provided an indication of the evidence that is available. By searching publicly available websites in addition to the published literature, it also allowed a comparison of the information available from formal (published literature) and informal (websites and related Web-based information) sources, and an exploration of the potential value, feasibility, and challenges presented by such an approach to searching for evidence.

## Methods

### Overview

The overall approach is based on that of a scoping review or study, a framework for which was provided by Arksey and O’Malley [20]. The following elements of the framework, in particular, informed the approach: identifying all literature regardless of study design, redefining search terms as familiarity with the literature increases, and using an iterative process. However, our methods were also guided by our aim to explore information available on websites about real-world projects or services and compare this with the published literature.

### Search Strategies

Because of the broad scope and varied terminology associated with social prescribing, and the intention to explore the gray literature in additional to conventional databases, we used a range of search strategies and an iterative approach. We searched the Cochrane Library, MEDLINE, PubMed, and Google scholar, and various sources of gray literature (Google, Yahoo, Bing, greylit.org, Opengrey.eu, and specific NHS websites such as NHS Evidence).

The initial search for published studies combined the following thematic fields to identify social prescribing projects: (1) social prescri* OR refer*; (2) program OR treatment OR management OR education OR support OR physical exercise OR aerobic OR physical activity OR leisure-time OR exercise OR sport OR leisure activit* OR physical fitness OR training OR physical performance OR weight loss OR weight reduction OR BMI OR body weight OR body mass index OR obesity OR overweight OR adiposity OR smoking OR tobacco OR cigarette OR social support OR loneliness; (3) diabetes; (4) primary care OR GP OR general practitioner OR community OR voluntary. In addition, we performed a search on lifestyle intervention trials for type 2 diabetes in MEDLINE and searched the 200 most recent hits for studies on social prescribing to test the effectiveness of our search strategy.

The search for gray literature (websites and related Web-based material) was based on the combination and iterative modification of the terms “social prescribing,” “social prescription,” ”primary care,” ”diabetes,” ”referral,” and “community services.” For instance, we searched the first 100 hits in the search engines for “social prescribing” and “diabetes.” If we found NHS websites with links or references to other projects of potential relevance, we followed these; otherwise we modified the search terms and repeated the process multiple times. An initial set of searches was conducted in December 2014.

The aforementioned searches were repeated in October 2016. At this point, further searches were conducted. Three search strategies for databases were developed based on the results of previous searches including further terms for link workers and “social prescribing interventions” and a term for prediabetes based on the rationale that the interventions would be of a similar nature to those for type 2 diabetes. These strategies are presented in Multimedia Appendix 1. The final search was a further Web-based search via the Google.co.uk search engine using the strategy “social prescribing” AND diabetes AND evaluation. The inclusion of evaluation as a term in this search was due to the fact that in the interim since the previous search, the use of the term social prescribing on websites had become much more extensive. Therefore, it was necessary to focus on identifying projects that had been evaluated. Searches for each of the individual projects located on websites were also carried out by searching using the project name on PubMed.

### Selection Criteria

Various definitions of social prescribing have been proposed, but it is clear that there is still a lack of consensus on its scope and interpretation. Therefore, a broad, pragmatic set of criteria was used to identify studies or projects that were likely to fit within the spectrum of relevant interventions: located in the United Kingdom or Ireland and involving a primary care provider referring patients including those with type 2 diabetes or prediabetes to a third party that is delivering nonmedical services in the community. Studies or evaluations had to include a description of the referral process or be described as a social prescribing service and mention type 2 diabetes or prediabetes either in the inclusion criteria or in the report or publication. In line with the aim of a scoping review, all study designs used in evaluation were included as were all forms of report. There was no formal language restriction, but studies and projects were excluded if they were available only as abstracts, protocols, located outside the United Kingdom or Ireland, or did not provide details regarding the process of referring the patients from primary care to the external health care, community, or voluntary service. The searches were conducted by 2 authors (ML and KP). Initial screening of each set of search results was carried out by 1 author, preliminary selections by each of the 2 authors were then compared to compile a “short-list,” full-texts were consulted, and final selections were made with reference to the third author (MP) where necessary.

### Data Extraction and Analysis

For each service, data were extracted by 1 of the authors (ML or KP) into a Microsoft Excel spreadsheet, documenting the name of the service, the name of the organization that carried out the service, the type of organization, the location, the criteria for referral, the type of intervention, outcomes, or objectives, the evaluation of the projects and its findings, the type of publications, and the terminology used in the project regarding social prescription. All extracted data was checked by at least one other author (KP, ML, or MP). For those projects that had undergone an evaluation where the relevant published papers or Web-based report was located, details of the evaluation were extracted. As a range of study designs were used in evaluation and because the main aim was to scope the information available, full appraisal was not carried out, as is the usual approach at this stage [21]. To provide an indication of the quality of the evidence on effectiveness, the following items were extracted: study design (including whether randomized and, where relevant, the control interventions employed), sample size, data collection methods, and outcomes measured. For this purpose, the main study or latest evaluation report was accessed. Details of any additional or related studies are discussed in the text.

## Results

### Search Results and Study Characteristics

**Figure 1:**
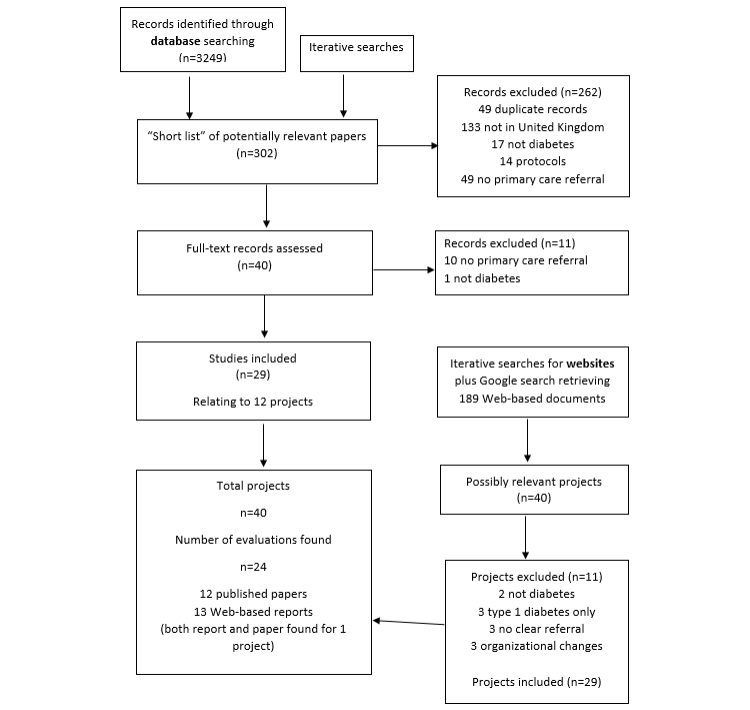
Process for the identification, screening, and selection of projects and evaluations.

A total of 40 projects or services and 24 evaluations were identified through the various searches and sources. A total of 3249 records from databases were screened, and a “shortlist” of 302 potentially relevant studies was compiled. After further screening and exclusion of studies, 29 papers related to 12 projects were selected, which met the inclusion criteria. For the Web-based searches, in addition to iterative searching, 189 documents retrieved by the final Google search were accessed, resulting in a list of 40 possibly relevant projects, of which 11 were excluded (2, not diabetes; 3, only type 1 diabetes; 3, no clear referral from primary care; 3, complex change interventions including but not focused on social prescribing). Twenty-nine projects met the inclusion criteria, with evaluations found for 13 projects (a Web-based report and a published paper were found for 1 project). Three additional project evaluations were located that included people with long-term conditions but, as diabetes was not specifically mentioned, these were not included. The screening process is shown in Figure 1.

Summaries of the evaluated projects or services focusing on the key study or report in each case are shown in Table 1. Details of the additional projects located on the Internet are presented in Table 2. The references to papers, reports, and websites are included for each project or service.

**Table 1:** Evaluated projects or services involving nonmedical community-based interventions.

Project or service	Intervention	Design of study or evaluation	Participants and setting	Outcomes and findings	Source
Addition-Plus [22,23]	Individually tailored, behavioral change intervention delivered by lifestyle facilitators plus intensive treatment	RCT^a^ (compared with intensive treatment alone) Also 5-year follow-up	478 adults with T2DM^b^ referred by HCP^c^ at 34 general practices in Eastern England (239 per group)	Health behaviors and cardiovascular risk factors (physical activity, diet, and smoking status) improved but no significant difference between groups after 1 year (*P*=.36, *P*=.07, and *P=*.28, respectively)	PP^d^
Age UK’s Fit for the Future “Social Prescribing“ extension project [24,25]	Social prescribing project (health care professional referral and range of support)	Service evaluation (mixed methods for main project; surveys and qualitative interviews for this extension project)	305 people with LTCs^e^ from 3 areas across the United Kingdom completed pre-post questionnaires; (247 completed both time points) (diabetes 16% of baseline group)	At 3-month follow up: less significant improvements in mental wellbeing (*P*=.36), attitude to healthy eating (*P*=.16) and social networks compared with the main project that varied across the 3 areas but some positive changes for life satisfaction; attitude to physical activity; and exercising	WR^f^
Birmingham Exercise on Prescription [26-28]	Exercise referral intervention grounded in Self-Determination Theory (SDT)	Exploratory cluster RCT (compared with standard exercise referral alone) Also regression analysis of support	347 adults with risk factors for CHD^g^ including T2DM referred to an exercise scheme at 13 leisure centers in Birmingham (184 in SDT group; 163 in standard group); number with T2DM not reported	Primary outcome: Physical activity using the 7-Day Physical Activity Recall (7DPAR). Other outcomes: BP^h^, BMI^i^, general health and fitness, anxiety, depression, vitality, quality of life, and well-being Improvements in physical activity, quality of life, and well-being in both groups. Between-group changes only significant for anxiety at 6 months (*P*=.003); physical activity (*P*=.93); quality of life (*P*=.40)	PP
City and Hackney Social Prescribing Project [29-32]	Social prescribing project (referral by GP^j^ to social prescribing coordinators managed by a voluntary, community, and social enterprise service for personalized signposting)	Mixed methods evaluation with control group (23 GP practices in total; 6 control GP practices) (survey or in-depth interviews)	184 people with depression, anxiety, or T2DM supported by project based in a London borough (matched with 302 in control practices). Number with T2DM not reported	Reported no statistically significant change in health, well-being, anxiety, depression, or A + E visits due to the SP intervention at 8 months. Qualitative interviews revealed positive or extremely positive experiences	WR
Community Oriented Diabetes Education (CODE or CODET2) [33-35]	Structured self-management education program	Mixed methods pre-post evaluation (physiological tests, questionnaires, and semi-structured interviews)	401 adults with T2DM referred to 31 CODE programs across the Republic of Ireland; 392 completed baseline; 237 (60%) completed the post-program evaluation	No difference in HbA1c^k^ (*P*=.14) or cholesterol (*P*=.06). Significant positive change in weight loss (*P*<.001), Significant increase in empowerment (*P*=.047), knowledge (*P*=.01) and quality of life (*P*<.001) at 26 weeks	PP and WR
DESMOND Newly Diagnosed [36-47]	6-hour structured group education program delivered by trained educators	Multicenter cluster RCT (compared with usual care) Also qualitative studies, 3 year follow-up, and a cost-effectiveness analysis	824 adults with T2DM referred to trial from 207 general practices in 13 primary care sites in United Kingdom	No significant difference in HbA1c (*P*=.52). Significant difference in weight loss (*P*=.03), smoking cessation (*P*=.03), changes in illness belief scores (*P*=.001), and depression (*P*=.03) after 12 months post diagnosis, in intervention group	PP
DESMOND Let’s Prevent Diabetes [48,49]	6-hour group structured education program	Cluster RCT (compared with standard care) Also retrospective analysis	880 adults with prediabetes from 44 GP practices in Leicestershire (invited by GP)	Nonsignificant 26% reduced risk of developing T2DM in the intervention arm (*P*=.18). Significant improvements in HbA1c (*P*<.05), LDL cholesterol (*P*<.05), sedentary time (*P*<.01), and step count (*P*<.01) at 3 years	PP
DESMOND Walking Away from Diabetes [47,50]	3-h group-based structured education program with pedometer use	Cluster RCT (compared with information provision)	808 people at high risk of T2DM from 10 GP practices in Leicestershire (data on 571 (71%))	Increases in ambulatory activity (*P*=.006) and self-reported vigorous-intensity physical activity at 12 months. No differences between groups at 3 years (*P*=.52) or in cardiometabolic markers	PP
EDIPS (European Diabetes Prevention Study)-Newcastle [51-53]	Intensive behavioral interventions to promote dietary modification and increased physical activity (group sessions plus signposting to community physical activities)	RCT (compared with usual care) Also qualitative study and follow-up	102 people with impaired glucose tolerance (prediabetes) (51 per group) referred by GP to trial and then to community physical activities in Newcastle	Significant reduction in the risk of developing T2DM, RR^m^ 0.45 (95% CI 0.2-1.2). Interim benefit achieved but no significant differences in sustained change (more than 2 years) for other outcomes (change in physical activity, fat, fiber, and carbohydrate intake). Mean duration of follow-up was 3.1 years	PP
EXERT (Exercise Evaluation Randomised Trial) [54]	Exercise referral scheme	3-arm RCT (compared leisure center-based exercise program, an instructor-led walking program, and advice-only)	943 patients from GP practices in 1 London borough; 13% diabetes (number with T2DM not reported)	Changes in exercise behavior and cardiovascular risk factors; waist–hip ratio, BMI, body fat, fitness, lifestyle behaviors, health status, quality of life, and health service usage and cost. All groups improved with no consistent differences between groups	PP
Living Well, Taking Control program [55,56]	Community-based program for diabetes prevention and newly diagnosed T2DM	Service evaluation involving economic evaluation, pre-post evaluation with all participants, process evaluation plus RCT (ComPoD) compared against wait list control group)	223 participants with T2DM (32.8%), 448 with prediabetes (66.0%) in service evaluation, and 40 recruited to trial in Birmingham and Bristol at the point of preliminary report	Preliminary results only: 6-month follow-up data for 123 participants. Significant pre-post changes in diabetes risk factors: weight loss (*P*<.001); HbA1c (*P*<.001); fiber intake (*P*<.005), depressive symptoms (*P*<.001); general health state (*P*=.002)	WR
Newly Diagnosed Type 2 Diabetes Dietary Education Group [57,58]	Group education program	Pre-post questionnaire to assess knowledge	126 adults with T2DM referred to program by GPs in North Wales	Significant differences in percentage of correct answers pre-post (*P* values <.01 for 9 of 11 questions)	WR
Newcastle Social Prescribing Project or NESTA People Powered Health project [59]	Social prescribing project (referral by primary care staff to nonclinical community services and networks plus information resource)	Mixed methods service evaluation (consultation with key stakeholders; assessment of project information and data; interviews with the health care professionals and patients)	124 people with LTCs referred from 6 organizations. Diabetes mentioned in cases only	Evaluation was based on numbers of patients achieving their individual goal (mainly health-related) and views of patients and HCPs on the service. Findings were used to inform a larger-scale project (ongoing).	WR
New Life New You [60-63]	Community-based lifestyle intervention; self-referral and signposting from primary care	Mixed methods pilot study (uncontrolled before-and-after study design with embedded interviews). Also interviews with black and minority ethnic community	218 people with impaired glucose tolerance (prediabetes) referred to project in Middlesbrough	Beneficial changes in physical activity, weight and waist measurements, and Finnish Diabetes Risk Score (FINDRISC) at 12 months. Follow-up with 134 (61%) participants	PP
PoLLeN (People, Life, Landscape and Nature) Bromley By Bow [64]	Social and therapeutic horticulture project	Service evaluation (mixed methods approach with validated outcomes questionnaires, feedback, interviews, and case studies)	39 adults with mental distress and conditions including diabetes referred to program in a London borough. One case study mentions diabetes	Short form of Clinical Outcomes in Routine Evaluation questionnaire (CORE10) and SWEMWBS^l^. Varied numbers of participants completed questionnaires at 3 time points. No significant changes pre-post. Qualitative data from clients showed appreciation for the project	WR
Ramadan Education and Awareness in Diabetes (READ) [65]	Ramadan-focused education program delivered by ethnic-speaking HCP and community link worker	Retrospective analysis of 2 groups (A and B)	111 Muslim adults with T2DM in Brent, London (Group A: 57 people referred or self-referred to and attended program. Group B: 54 invited, did not attend)	Significant differences in weight loss (*P*<.001), and hypoglycemic events (*P*=.001), which were sustained in group A at 12 months	PP
Rotherham Social Prescribing service [66-68]	Social prescribing project (individual advice, signposting service from Voluntary and Community services (VCS) advisors)	Service evaluation (monitoring data, interviews, case studies, and surveys)	1,607 people with LTCs referred to the service. Diabetes only referred to in case studies	Reduction in demand for hospital care, improvements in well-being, and social impact were reported based on estimates from a subset of beneficiaries and cost-saving estimated	WR
Rugby Social Prescribing Project: ConnectWELL [69]	Pilot social prescribing project in 4 GP surgeries to support and signpost individuals to services and activities in the local and voluntary community	Service evaluation aimed to produce statistical evidence and recommendations for other social prescribing initiatives	People in Rugby with various health problems, for example, diabetes. Number with T2DM not mentioned	Interim report located online but publication status unclear. Anticipate data reporting in full report (as yet not found). No T2DM specific-outcomes reported	WR
Sadee Smile (South Asian Diabetes Education, Empowerment and Self-Management in Leeds) [70]	Pilot education program led by nonclinical tutors	Service evaluation of 11 courses (pre-post) including knowledge questionnaire, focus groups, case studies, and interviews with staff	113 adults with T2DM referred to program in Leeds	Knowledge, skills, and confidence assessed around diabetes management improved post service. Increased physical activity reported	WR
South Gloucestershire Exercise on Prescription [71-72]	Tailored, supervised exercise referral scheme. Patients with LTCs referred by GP	Pre-post comparison of physiological data, WEMWBS, demographics on participants, and usage data; cost data; interviews with staff and patients	2505 participants referred to scheme. Number with T2DM: 21.9% of males and 9.9% of females.1379 (55%) considered completers	At 12-week follow-up: Significant decrease in systolic BP (*P*<.01), waist (*P*<.03), number of 30-min physical activity sessions and well-being (*P*<.001). No significant difference in weight (*P*=.63), BMI (*P*=.25), hip (*P*=.86), or diastolic BP (*P*=.56)	WR
Well UK South West Well-being or South West Well-being (SWWB) [73,74]	Social prescribing project (including a portfolio of initiatives)	Service evaluation (longitudinal study using outcome measures, surveys and interviews, case studies)	737 people with low-level mental ill health, approaching older age, and families on lower incomes referred from Bristol area. Case studies mention diabetes	Positive changes in general health, physical activity, diet, mental well-being, and social well‑being based on self-reporting	WR
Wigan and Bolton Exercise Referral Scheme [75]	Exercise referral scheme plus information	RCT (compared with information alone)	545 sedentary adults with risk factors for coronary heart disease including diabetes from 46 of 52 general practices in one borough in northern England (275 intervention, 270 control). Number with T2DM not reported	Primary outcome: moderate or vigorous activity for 90 min per week: significant improvement at 6 months (*P*=.05) but not significant at 12 months (*P*=.18) based on 7-Day Physical Activity Recall (7dPAR)	PP
Wirral Lifestyle and Weight Management program [76]	Intensive 12-week program consisting of a variety of group meetings or tailored one-to-one sessions	Economic analysis (preliminary report)	3810 people with obesity with comorbidities of T2DM or CHD. Number with T2DM not reported	Majority of subgroups showed significant reductions in weight at 12 weeks (*P*<.001). Limited data available for all other outcomes except BMI. Associated cost savings for T2DM calculated	WR
X-PERT [77-79]	Patient-centered, group-based self-management program (6 2-hour sessions)	RCT (compared with individual appointments)	314 adults with T2DM in 3 boroughs in northern England (157 per group)	Significant improvement in X-PERT group compared with control for HbA1c, weight, BMI, waist circumference (all *P*<.001), total cholesterol (*P*<.01), self-empowerment (*P*<.04), knowledge (*P*<.001); also in physical activity, foot care, fruit and vegetable intake, enjoyment of food at 14 months	PP

^a^RCT: Randomized controlled trial.

^b^T2DM: type 2 diabetes.

^c^HCP: Health care practitioners.

^d^PP: Published journal paper.

^e^LTC: Long-term conditions.

^f^WR: Web-based report.

^g^CHD: Coronary heart disease.

^h^BP: Blood pressure.

^i^BMI: Body mass index.

^j^GP: General practitioner.

^k^HbA1c: Hemoglobin A1c.

^l^(S)WEMWBS: (Short) Warwick Edinburgh Mental Wellbeing Scale.

^m^RR: Risk ratio.

**Table 2:** Additional projects or services identified from Web-based searches.

Name of project or service	Location	Referral process	Types of patients	Type of service, what is offered and by whom	Aim of service
Altogether Better Diabetes^a^[80,81]	Northern England (Leeds, York)	Community Health Champions (CHCs) based in GP^b^ practices can refer to GP or to community	Diabetes	CHCs signpost clients to activities, accompany clients, and provide networking opportunities	Improving the health and well-being of communities
Be active plus [82,83]	Birmingham (South and Central)	GP or nurse refers patients at surgery to program	Different conditions including diabetes, hypertension, and obesity	Individually tailored exercise program plus support from Health and Fitness Advisors at local leisure center	To increase the amount of physical exercise
Building Health Partnerships: Bristol [84]	Bristol	Referral to Public Health Improvement Teams	Black and minority ethnic diabetic (primarily Somali and Asian people)	Individual support from Public Health Improvement Teams, cooking events	Healthier lifestyle
Camden Exercise Referral^a^ [85-87]	London (Camden)	GP or health professional refers to Active Health team	A range of conditions including diabetes	Individualized exercise program	Active lifestyle changes
Diabetes Education and Revision in East Kent (DEREK) [88]	East Kent	GP or practice nurse refers patients to education coordinator	Type 2 diabetes	Education program at various venues	Better management of diabetes, shared experiences, better relationships with health care professionals
Diabetes Education Awareness for Life (DEAL)^a^ [89]	Berkshire	GP or practice nurse or community nurse refers patient to program	Newly diagnosed and existing type 2 diabetes	Education program (also well-being groups for talking therapy) in community	Healthier lifestyle, education
Go4life [90-92]	North Somerset	Referral to volunteers that offer 1:1 support for up to 6 weeks	Type 2 diabetes	Individual support and education	Healthier lifestyle, specifically meeting physical activity goals
Good2go [93]	York	GP or practice nurse refers patient to program	Type 2 diabetes	Education program	To better manage the disease
HARRIET (Harrogate Initiative for education in type 2 diabetes) [94,95]	North Yorkshire and York	GP referral	Type 2 diabetes	Education program	Not specifically stated (general aim: to help people become experts on managing their condition’)
Juggle Diabetes Education Service [96]	Nottingham	GP refers patient to program delivered in variety of venues (or can self-refer)	Type 2 diabetes (not treated with insulin)	Education program	To better manage the disease
King’s Lynn diabetes type 2 education program [97]	King’s Lynn	GP or practice nurse referral	Type 2 diabetes	Education program	Not specifically stated
Life and health with diabetes [98,99]	Buckinghamshire, Bedfordshire, Berkshire, Hertfordshire, Oxfordshire, and Uxbridge.	GP referral	Type 2 diabetes	Education program	Improve understanding of diabetes, confidence in self-management and quality of life
Living with diabetes [100]	Bristol	GP refers patient to program	Newly diagnosed type 2 diabetes (within last 12 months)	Education program	Healthier lifestyle, education
Newham Community Prescriptions [101,102]	London (Newham)	Referral by GP to community prescription navigators	Type 2 diabetes and patients at risk	Personalized information and support service by community prescription navigators, physical activities offered by VSCE partners, gardening	Adherence to physical activity
Social prescribing project^a^ [103,104]	Cullompton, Devon	GP refers patients to health facilitator who provides advice on exercise, diet, and so forth	Patients with CVD, type 2 diabetes, prediabetes, and other diseases	Individual advice, signposting service	Support patients to exercise and socialize
Start-Up exercise referral scheme^a^ [105,106]	Cambridge	GP, practice nurse, or health professional refers to scheme	People with a range of medical conditions including diabetes	Exercise programs and support, assistance, and supervision from specialist exercise professional	Support patients whose health would benefit from leading a more active lifestyle

^a^Projects that are currently undergoing evaluation have been evaluated as part of a larger-scale evaluation or where the evaluation report was not available.

^b^GP: General practitioner.

### Overview of Projects

The included projects represented a range of initiatives and interventions and were commissioned by a range of organizations, either solely or in collaboration, including NHS organizations, local government, and charities. Three projects had a national coverage, providing a standardized program (eg, DESMOND) [47] or a service that was locally adopted from an initial version (eg, X-PERT) [79] or a “social prescribing” intervention provided as part of a national program [24]. The remaining projects were limited to a local area, ranging from city districts to counties or regions.

### Locating the Relevant Information

Although all published studies were located using a standard medical database, information on the projects or services was found from a variety of sources. Much of the information was retrieved from websites of the organizations delivering the projects. However, in order to include information on the prespecified aspects and, where evaluation reports were not available, it was necessary to follow links to other resources or websites and to access presentations, minutes of relevant meetings, and reports. The specific sources used are referenced in Table 2.

The studies published in peer-reviewed journals met our broad definition of social prescribing as a referral process from primary care to an external provider of nonmedical services in the community. These studies were not, however, described as social prescribing projects. In fact, only 1 study was located on PubMed that referred to social prescribing [107]. It was potentially relevant, as it was focused on long-term conditions. However, diabetes was not mentioned, and therefore it was not included. The descriptions varied considerably across the projects. Some were referred to as educational programs emphasizing the type of intervention, as “diabetes programs” emphasizing the condition, or GP referral programs focusing on the process. Projects or services were also described as a “community prescription,” a community service, a voluntary, community, and social enterprise undertaking, or a social prescribing project.

### The Range of Interventions

The intervention in the majority of projects (n=16) was a structured group education program that included multiple thematic areas such as disease information, disease management, healthy lifestyle, and health consequences of diabetes. For instance, the DESMOND program is a structured education program over 6 hours on 1 or 2 days [47]. In the X-PERT diabetes program, participants receive a handbook and are taught by trained educators about diabetes, weight management and other lifestyle factors, and diabetic complications [77]. A number of services involved referral or recommendation of exercise; 7 schemes were based solely on exercise (“Exercise on Prescription” schemes), whereas others offered access to exercise classes as part of a “menu” of community activities. For example, 1 service offered free physical activity options (eg, Tai Chi, Zumba, Community Gym) for 12 weeks at maximum or an individually tailored exercise program for 12 weeks in combination with personal support, a final meeting, follow-up contacts, and a report that was sent to the GP [101].

Of the interventions that did not involve group education or exercise on prescription, 1 involved trained and quality-assured lifestyle facilitators delivering an “individually tailored behavior change intervention” [22]. Another provided individual support to black and minority ethnic people with type 2 diabetes via “Public Health Improvement Teams” [84]. Other interventions included community-based diabetes prevention programs [48,50,60], a lifestyle and weight management program [76], and a social and horticultural project [64]. The remainder were described as “social prescribing.” In one of these, a personalized signposting service was provided by “Social Prescribing Coordinators” to local services provided by a voluntary, community, and social enterprise (VSCE) group [30]. Similar services were provided by Community Health Champions, Community Prescription Navigators, Social Prescribers or Social Prescribing Workers (or VCS Advisors), link workers, and a practice-based health facilitator (or health manager).

### Evidence from the Evaluations

We identified a variety of types of research and evaluation on the interventions, ranging from local projects with regular audits using questionnaires, to national programs tested using multicenter randomized controlled trials (RCTs). Of the 24 evaluations, 10 involved RCTs of behavioral, education, or exercise interventions. The majority were service evaluations that used a combination of approaches, both qualitative and quantitative. This required collection and analysis of monitoring data, measurement of outcomes using validated measures or self-reporting, cost-effectiveness calculations, and methods for gaining an insight into the perspectives of both providers and users of the services through surveys, interviews, and focus groups.

Of the education programs, the DESMOND program, one of the most extensively tested, had undergone a series of clinical trials and other evaluations. These include a large-scale RCT with a subsequent 3-year follow-up study [36,44], a more recent nonrandomized trial comparing health care professional and lay educators [45], and a study on cost-effectiveness [42]. The results revealed that its group education program for patients with newly diagnosed type 2 diabetes was effective at improving some key outcomes but not all. There were positive improvements in illness beliefs, which were still sustained after 3 years, but differences in biomedical or lifestyle outcomes were not apparent after this period [44]. Further research revealed that there was no difference in study outcomes between patients who were referred to 2 health professional educators and those who were referred to a team consisting of a professional educator and a layperson [45]. The cost-effectiveness analysis showed DESMOND to be cost-effective compared with usual care at 12 months [42]. Further studies focused on the facilitators and their skills, and the perspectives of participants in the program, through the use of qualitative approaches [39,46].

A second education-based intervention, X-PERT, has also been tested in an RCT, and a range of benefits were reported on physiological and behavioral outcomes, including improved glycemic control, BMI, diet, and self-management skills at 14 months [77]. A noncontrolled trial of an adaptation of the program designed for Bangladeshi adults with type 2 diabetes, which did not involve GP referral and therefore is not included in the tables, has also been completed but was underpowered due to low attendance rates [108,109]. A program designed specifically for Muslim diabetics was also assessed by retrospective analysis and reported beneficial effects [65].

Pre-post questionnaires and physiological measures were used to assess the CODE (Community Oriented Diabetes Education) program with positive trends but not significant changes in physiological measures. However, there was a significant increase in participants’ knowledge scores, coping ability, motivation to change, and making informed decisions about their diabetes [33].

Other studies investigated the effectiveness of exercise on prescription schemes [26,54,71,75]. Mixed results were reported with effects, if observed, either limited to reduction of anxiety or not maintained over time. One RCT found no difference between an exercise program, instructor-led walking, or advice only [54], while a regression analysis of one scheme explored how the effects of support differed as a function of who provided the support [27]. RCTs were also used to assess the effects of interventions described as behavioral or lifestyle change interventions [22,51,60].

Internal or external evaluation of services described as social prescribing, either ongoing or completed, was reported on a number of the websites. Of these, evaluation reports were found for 6 “social prescribing” services. In other cases, the results of the evaluations were reported in brief on the website, or in the minutes of relevant meetings or other documents. Some services had been evaluated as part of large projects, whereas in several cases, reports were unavailable or could not be located. One further service had been approved by QISMET (the Quality Institute for Self-Management Education and Training), an independent body developed to support self-management providers and commissioners [98]. In some cases, the collection of data was reported but the results were not located by the searches we performed.

The type of evaluation ranged in design. Basic numerical data were collected including numbers referred to and accessing the service, and numbers of people reporting changes in various aspects such as increased physical activity or simply completing the program. Questionnaires and more complex designs, for example, mixed approaches using questionnaires combined with interviews or objective outcome data, were also employed in some cases.

One social prescribing project in inner London that underwent a full evaluation using a mixture of qualitative and quantitative methods also incorporated a matched control group [29]. No significant differences were found between groups based on the quantitative measures, although the qualitative data indicated beneficial effects with positive responses from the participants.

In the case of the other projects described as social prescribing for which full evaluations were found, a pre-post design was used [24,59,66,73]. Although positive outcomes were reported in, for example, physical activity, weight, blood pressure, mood, and social outcomes, (see Table 1 for the range of outcomes reported), this design did not allow firm conclusions on effectiveness to be drawn. One evaluation reported reduced hospital admissions and improved social outcomes [66]. Several evaluations focused on aspects related to implementing such a project in practice by exploring the perspectives of the patients, the health care providers, the role of the link worker, and the project governance to conclude with recommendations for future social prescribing projects [59].

While a formal, in-depth systematic appraisal of the methodology was not carried out, a number of issues were encountered when extracting data from the evaluations. It proved difficult in some reports to find the rationale, or occasionally the actual numbers, for the samples used in evaluations. It was also clear that missing data was an issue, particularly where follow-up was at several time points. This is, perhaps, not surprising considering that the aim of social prescribing and related interventions is to direct the patient to the community and voluntary sector rather than encourage follow-up in primary care. However, what was not always clear was how missing data were treated. Finally, as described earlier, referral was often based on psychological, social, or practical needs; this meant that the primary diagnosis was not often reported nor were the results provided by diagnosis.

## Discussion

### Principal Findings

Our study revealed limited evidence on social prescribing, specifically for type 2 diabetes in the United Kingdom and Ireland in peer-reviewed literature. Only 1 published study referred to social prescribing and that involved people with long-term conditions with no specific mention of type 2 diabetes. Nevertheless, broader Web-based searches demonstrated the existence of numerous “real-world” projects that investigated potentially relevant community-based interventions and had undergone evaluation using a variety of methods. Analysis of these projects also revealed the diverse ways in which the concept of social prescribing has been implemented in practice. The findings of this scoping study do not allow firm conclusions as to the effectiveness of social prescribing for people with type 2 diabetes. The results of the broader searches provide a clearer picture of the potential effects, possible outcomes, and processes involved, and thus inform research in this area. These evaluations also have the potential to provide valuable information for those organizations planning to implement similar services in future. This study has also revealed the different ways in which social prescribing may be described in practice, and this will aid those searching for evidence in future.

### Challenges and Limitations

The challenges encountered when designing and conducting this study have led to a number of limitations. First, we attempted to identify evaluations of what is effectively a process, the referral of patients from primary care to a nonmedical, community-based activity, program, or service, rather than an intervention per se. In addition, some studies may not have referred specifically to social prescribing and described the intervention using different wording due to the lack of consensus on terminology in this area, thus precluding their identification. We attempted to address these points by using a range of several search strategies and an iterative process whereby we amended the later strategies according to the results of the initial searches. Although we extended the later database searches by including terms for prediabetes and a range of interventions, because of the recent proliferation of websites on the prevention of diabetes, we did not search specifically for diabetes prevention programs on the Web. Such programs, for example, those launched as part of the national Diabetes Prevention Program [110], are likely to use similar models and interventions to the projects included in this review, but further exploration of this aspect would be valuable. The selection of studies also relied on the referral process being reported clearly. The complexity of both identifying and assessing the evidence on social prescribing is demonstrated by the case of the X-PERT project. The RCT on X-PERT was initially excluded from our search because it did not include referral from primary care to an external service provider, whereas in practice, patients are referred from the general practice to the X-PERT educators via social prescribing. A similar situation occurred with later trials of the DESMOND program. Related to this point, it is debatable whether referring patients to a program as part of an RCT is adequately replicating the social prescribing process in practice. A conventional systematic review of published literature might, therefore, not represent a feasible approach to identify the entire literature and assess the effectiveness of the process.

A second potential limitation was that we limited the search to the United Kingdom and Ireland. The heterogeneity of national health systems, the plethora of diabetes websites, and the varied language used in describing this concept outside the United Kingdom would have proved a considerable challenge, had we attempted to cover the worldwide literature. This does mean that broadly similar projects in other countries were omitted from this review. Third, only information available on the Internet was retrieved and there was no direct contact with those delivering programs or services for further information, confirmation, or clarification. For this reason, it is possible we might have omitted relevant projects or incorrectly interpreted the information we were able to locate. Website information also evolved throughout the course of the study. Finally, by focusing on diabetes and including only those programs or services that specifically referred to people with this condition, we might have omitted relevant services or programs. Conversely, using a broad definition of the intervention resulted in the inclusion of projects and services that, perhaps, do not fit with the original concept of social prescribing as being a mechanism to refer from primary care to the voluntary and community sector. In some cases, the services, although nonmedical and community-based, were still provided by health professionals.

Nevertheless, our broad searches have demonstrated that social prescribing is prevalent in the United Kingdom, and that information is available that is potentially valuable but not currently in the published literature and therefore difficult to locate and access. At this point, our analysis on the range of projects and services, and the evaluations of these, may prove helpful.

**Figure 2:**
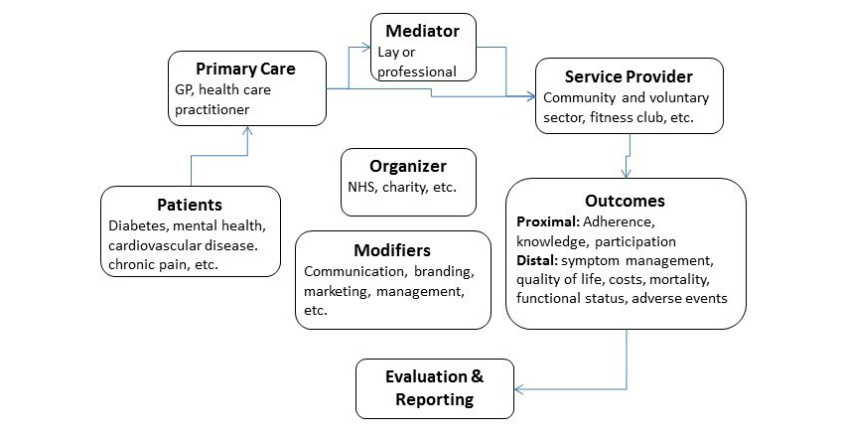
A model of social prescribing including people with type 2 diabetes.

### The Social Prescribing Concept and Terminology Surrounding This Concept

It is clear from the findings of this review that different models have been implemented in practice, some of which fit more closely with the original concept of “social prescribing.” It is also clear that although the term “social prescribing” is more widely used in practice in the United Kingdom, it is not a concept that is currently recognized in the main medical databases or in other countries. Even within the United Kingdom, the link workers, who are a crucial part of many social prescribing services, are described in different ways. The findings from this scoping review do, however, suggest a preliminary model for social prescribing such as that presented in Figure 2. This will require further development and refinement.

### Translating Research Into Practice

It has been argued that the term “translational research” does not distinguish between testing of new treatments and research on how to implement new treatments in practice [10]. This is supported by our finding that the process of social prescribing, potentially the crucial step in the implementation process, is neglected in comparison to the intervention itself. A wide range of models of the process were revealed, for example, direct referral by the GP to the community sector, referral to the community via an in-practice link worker, or referral to a link worker based in the voluntary and community sector. Only 1 study attempted to address whether the process of prescribing an intervention had any influence by comparing referral to exercise programs in the community with tailored advice and information on local exercise facilities. None of the evaluations we located investigated, for example, how direct referral by a GP compared with referral via a link worker.

In general, many barriers exist that hamper the adoption of research into diabetes into clinical care at the level of the patient, primary care, health care system, community, and society [111]. Several countries have developed diabetes prevention programs for clinical care [112], whereas there has been limited translational research in the United Kingdom [113]. The effects of lifestyle programs in real-world primary care for patients with prediabetes or T2DM are small [114] and depend substantially on the program and its implementation [115]. Development, evaluation, and reporting of translational research programs need to be adapted to reflect the context and consider generalizability [116]. In exploring the effectiveness of implementation, various measures of success need to be taken into account and interpreted. For example, although in many cases, qualitative data in the evaluations found in our searches revealed a positive patient experience, this has not yet been supported by quantitative outcomes to the same degree. It is also clear that, in evaluating the effectiveness of these programs, a range of generic outcomes are used and the focus is often not on disease-specific indicators such as hemoglobin A1c. This is because social prescribing grew out of a perceived need to address broader social, behavioral, and practical issues that impact on, and may be caused by, the disease. Thus, the focus is on the person who has diabetes rather than on the disease itself. Interest in social prescribing is increasing, and its effects on specific groups such as those with diabetes are likely to be of interest, making it important to provide guidance on how real-world evaluations should be conducted and reported to ensure that the data collected can be more accurately compared and analyzed. The results of our analysis of real-world project evaluation will inform this guidance, while guidance such as that produced by Diabetes UK on commissioning to involve nontraditional providers in the support of people with long-term conditions is also valuable [117].

Our review also suggests a need to improve the reporting of implementation research, specifically service evaluations. Social, cultural, legal, demographic, and other factors that are controlled in trials become relevant influences during the process of implementation [118]. Combining these factors with the diversity in settings, methods, and outcomes of the real-world projects indicates that further development of existing reporting guidelines would be beneficial [119]. However, although standardization of reporting of evaluations would allow greater comparison across services and projects, a service evaluation is produced for a specific organization and to meet the specific requirements of that organization. Thus, increasing standardization of reporting, should this be considered feasible, must be balanced with the need to meet these requirements.

It has been suggested that evidence synthesis plays a pivotal role in developing or refining a framework of translational research [120]. A first step to enable this would be to better understand and define the multiple dimensions of translational research including implementation projects in real-world settings [10]. This might begin with a debate on reporting of different types of translational research, such has been proposed for Web-based health interventions [121]. Additionally, the challenges encountered in identifying relevant projects in this study indicate that it might prove fruitful to develop specific databases or networks to support larger-scale and more rigorous evaluation and comparison of programs and their relative cost-effectiveness.

Finally, a discussion might be necessary on how the principles of systematic reviews of health interventions can be applied to translational research, as the hierarchical model of evidence-based medicine has been shown valid only for certain questions such as efficacy [122]. It has been suggested that the study design alone may be an inadequate marker of the quality of evidence for interventions that are complex, program based, and dependent on context [123]. Consideration must be given to the evaluation process and its ability to detect effects and distinguish between failure of an intervention and failure of the delivery process. It is clear from this review that approaches incorporating a range of methods are necessary for evaluating the implementation of such complex interventions in practice. Novel approaches to synthesizing the evidence in this field are already underway [124].

As part of this discussion, it will be worth considering what role service evaluations of real-world projects play in the evidence base, and whether there are minimum standards for conducting such evaluations. Carrying out formal quality appraisal of these is complex, as they involve a range of data collection approaches, and it is unclear how best to accurately assess and compare the methodology used in individual evaluations. Although standardized tools and checklists exist for designs such as RCTs and qualitative studies, similar tools are not widely available for complex designs. As described earlier, there is also a question of how service evaluations may best be identified, as it is clear that the majority remain in the gray literature and would not be located via conventional systematic search techniques.

### Conclusions

This review revealed the range of models of social prescribing and related nonmedical community-based services, and the extent of evaluation that has been carried out to assess the potential benefits of these, which would not have been achieved by searching databases alone. Although the evidence from these evaluations does not prove that social prescribing is an effective measure for implementing nonmedical interventions for patients with type 2 diabetes in the United Kingdom, there is sufficient to indicate the value of further evaluation and comparison, particularly if focused on real-world settings. The findings of this scoping study may inform future evaluation. Further research is necessary to better understand the communication between primary care and community or voluntary sector and to improve the documentation of the final step of translational research, implementing interventions in real-world settings.

Accessing Web-based information provides a potential method for investigating how specific innovative health concepts, such as social prescribing for type 2 diabetes, have been implemented in practice and the full extent of the evaluation of such innovations. Several challenges were encountered including defining the concept, focusing on process plus intervention, and systematically searching varied and evolving Web-based sources. Obtaining sufficient relevant information requires searching for and analyzing information from a range of sources. The methods and findings from this study have already informed a broader scoping exercise on the evaluation of social prescribing projects in the United Kingdom, which is underway. Further exploration of this approach will inform future research on the application of health-related concepts into practice.

## Appendix

Multimedia Appendix 1 Search strategies.

## Abbreviations

BMI: body mass index
BP: blood pressure
CHD: coronary heart disease
GP: general practitioner
HbA1c: Hemoglobin A1c
HCP: health care practitioners
LTC: long-term conditions
PP: published journal paper
RCT: randomized controlled trial
RR: risk ratio
(S)WEMWBS: (Short) Warwick Edinburgh Mental Wellbeing Scale
T2DM: type 2 diabetes
WR: Web-based report
