# Supplementary material for: Searching for Real-World Effectiveness of Health Care Innovations: Scoping Study of Social Prescribing for Diabetes
Source: J Med Internet Res. 2017 Feb 2;19(2):e20. doi: 10.2196/jmir.6431 (PMC5314100; doi:10.2196/jmir.6431)
Supplement: Multimedia Appendix 1 [file jmir_v19i2e20_app1.pdf]

Initial search: carried out in 2014 and repeated in October 2016 (MEDLINE)

(program OR treatment OR management OR education OR support OR "physical exercise" OR aerobic OR "physical activity" OR "leisure-time" OR exercise OR sport OR "leisure activit" OR "physical fitness" OR training OR "physical performance" OR "weight loss" OR "weight reduction" OR BMI OR "body weight" OR "body mass index" OR obesity OR overweight OR adiposity OR smoking OR tobacco OR cigarette OR "social support" OR loneliness) AND ("social prescri*" OR refer*) AND diabetes AND ("primary care" OR GP OR "general practitioner" OR community OR voluntary)*

Additional strategy focused on social prescribing interventions (MEDLINE)

(adult learning or aqua-therapy or arts or art class or art group or befriending or bibliotherapy or book group or book club or choir or computerised CBT or Community education group or community group or cycling or dance class or ecotherapy or eco-therapy or exercise on prescription or exercise on referral or exercise class* or fishing club or gardening or green activity or Green Gym or green prescription or group activities or guided walks or gym or health walks or horticulture or "knit and natter clubs" or Learning prescriptions or lunch club or museums in health or museums on prescription or music group or mutual aid or reading on prescription or self-help groups or signposting or swimming or team sports or time banks or volunteering or walking group or walkers group) AND (uk or united kingdom or great britain or wales or scotland or england or Ireland) AND (diabetes or pre-diabetes)*

Additional strategy focused on 'link workers' and social prescribing interventions plus types of designs used in evaluating complex interventions such as social prescribing (PubMed)

(cohort study OR cohort studies OR clinical trial OR randomised controlled trial OR randomized controlled trial OR multicentre study OR multicenter study OR qualitative research OR pilot projects OR pilot study OR focus group OR surveys and questionnaires OR evaluation project OR program evaluation OR evaluation report OR programme evaluation) AND ("Community Health Workers"[Mesh] OR "health facilitators" or "health facilitator" or "health trainer" or "health trainers" OR "social prescribing" or "social prescriber" or "link worker" or "health adviser" or "health advisor" or "community navigator" or "care navigator" or "health champion" or "referrals facilitator" or "lifestyle facilitator" or "fitness adviser" or "fitness advisor" or "peer educator" or "lay educator" or "prescription navigator" or signposting OR gardening or "lunch club" or "book group" or "book club" or "walking group" or "walkers group" or horticulture or "art class" or "art group" or yoga or "music group" or choir or "dance class" or "green activity" or gym or "community group" or "sports club" or arts or "healthy eating" or "cookery class" or befriending or volunteering) AND (uk or united kingdom

or great britain or wales or scotland or england or ireland) AND ("Diabetes Mellitus, Type 2"[Mesh] OR "Prediabetic State"[Mesh])

Additional search strategy focused on the process and terms reflecting the specific interventions likely to be used for type 2 diabetes i.e. lifestyle-related (PubMed)

(uk or united kingdom or great britain or wales or scotland or england or ireland) AND (link worker or health advisor or health adviser or health trainer or community care navigator or community navigator or health champion or social prescribing or social prescriber or community or voluntary) AND (physical exercise or aerobic or physical activity or leisure-time or exercise or sport or leisure activit or physical fitness or training or physical performance or diet* advice or weight loss or weight control or weight reduction or support or education or training or advice or social or physical exercise or aerobic or physical activity or leisure-time or exercise or sport or leisure activit* or physical fitness or training or physical performance or fitness or (lifestyle and community) or (lifestyle and advice) or (lifestyle and support)) AND (type 2 diabetes or "Diabetes Mellitus, Type 2"[Mesh])*
